# The effects of dialysis modality choice on cognitive functions in patients with end-stage renal failure: A protocol for systematic review and meta-analysis

**DOI:** 10.1097/MD.0000000000026209

**Published:** 2021-06-04

**Authors:** Tongbin Gao, Yongjian Ji, Yinghui Wang

**Affiliations:** Department of urology, Weifang People's Hospital, Shandong, China.

**Keywords:** chronic renal failure, end-stage renal disease, hemodialysis, peritoneal dialysis

## Abstract

**Background:**

There is no published meta-analysis comparing the effects of dialysis modality choice on cognitive functions in patients with end-stage renal disease . Therefore, we perform a protocol for systematic review and meta-analysis to evaluate cognitive function in peritoneal dialysis versus hemodialysis patients.

**Methods:**

This protocol is conducted according to the Preferred Reporting Items for Systematic Reviews and Meta-Analysis Protocol (PRISMA-P) statement guidelines. Related articles were identified by searching Web of Science, Embase, PubMed, Wanfang Data, Medline, Science Direct, and Cochrane Library. The risk of bias assessment of the included articles was performed by two authors independently using the tool recommended in the Cochrane Handbook for Systematic Reviews of Interventions. All calculations were carried out with Stata 11.0 (The Cochrane Collaboration, Oxford, United Kingdom).

**Results:**

The results of this systematic review and meta-analysis will be published in a peer-reviewed journal.

**Conclusion:**

We hypothesized that patients on peritoneal dialysis demonstrated a lower odd of cognitive dysfunction compared to those on hemodialysis.

**Open Science Framework registration number:**

10.17605/OSF.IO/NWCZK

## 1 Introduction

Chronic renal failure is an important public health problem that has become a global health concern.^[1]^ Chronic renal failure is defined as objective renal damage for at least 3 months or lowering the glomerular filtration rate below 60 ml/min/1.73 m^2^.^[2]^ It is emerging as a complex global health problem with a huge economic burden both on the affected family of patients and on the healthcare delivery system. Although different from one country to another, the global prevalence of chronic kidney failure is 242 in a million with an annual increase of 8%.^[3]^ With the growing world population, an increasing number of end-stage renal disease (ESRD) patients can be also predicted. Such patients are largely managed using peritoneal dialysis, or hemodialysis.

Cognitive dysfunction is a major consequence of renal dysfunction and has further detrimental effects on quality of life for ESRD patients.^[3]^ The reported prevalence of cognitive impairment among patients with ESRD, as assessed using neuropsychological tests, varies from 16% to 38%.^[4]^ Cognitive dysfunction in ESRD subjects can be attributed to several factors, including premature aging, dialysis-related complications and uremia itself, exacerbated by a multitude of renal failure-associated comorbidities such as renal anemia, hypertension, diabetes, malnutrition, vascular calcifications, cerebrovascular disease and bone-mineral disorders.^[4–7]^

Compared to hemodialysis, peritoneal dialysis is potentially a more physiologic form of renal replacement therapy.^[8]^ This is likely due to less hemodynamic fluctuations along with the nature of a steady 24-hour removal of uremic toxins yielding a better preservation of residual renal function in peritoneal dialysis patients.^[9]^ To the best of our knowledge, there is no published meta-analysis comparing the effects of dialysis modality choice on cognitive functions in patients with ESRD. Therefore, we perform a protocol for systematic review and meta-analysis to evaluate cognitive function in peritoneal dialysis versus hemodialysis patients.

## 2 Methods

### 2.1 Protocol registration

The prospective registration has been approved by the Open Science Framework registries (https://osf.io/nwczk), and the registration number is 10.17605/OSF.IO/NWCZK. The protocol was written following the Preferred Reporting Items for Systematic Reviews and Meta-Analyses Protocols (PRISMA-P) statement guidelines.^[10]^ Ethical approval is not necessary because this is a meta-analysis.

### 2.2 Study selection

Electronic databases including Web of Science, Embase, PubMed, Wanfang Data, Medline, Science Direct, and Cochrane Library were searched in May 2021 by two independent reviewers. The following search syntax was used in the advanced search engine of databases: ((“hemodialysis” AND (“peritoneal dialysis”)) AND (“cognitive” OR “cognition” OR “dementia” OR “memory” OR “function”)). The reference lists of the included studies were also checked for additional studies that were not identified with the database search. There was no restriction in the dates of publication or language in the search.

### 2.3 Inclusion and exclusion criteria

Studies were considered eligible if they meet the following criteria:

(1) randomized controlled trials
(2) study population of patients with ESRD
(3) intervention group received peritoneal dialysis and control group received hemodialysis.

Studies were excluded if they were available only as case reports, comments or letters, biochemical trials, protocols, conference abstracts, reviews or if predefined outcome data required for analyses were lacking.

### 2.4 Data extraction

Two investigators reviewed all the titles and abstracts independently. Data was extracted from eligible full-text studies. The data included study population, demographical characteristics, year of publication, country, age, gender, intervention regimens, duration of follow-up, and study outcomes.

Primary outcomes were cognitive dysfunction, defined as memory loss, perceptual motor disabilities, disturbances in executive functioning, attention and critical thinking. Secondary outcomes were dialysis-related complications and medical costs. Data extraction was performed independently, disagreements between the 2 reviewers were discussed and, if necessary, the third author was referred to for arbitration. If the data were missing or could not be extracted directly, authors were contacted by email.

### 2.5 Quality evaluation

The risk of bias assessment of the included articles was performed by two authors independently using the tool recommended in the Cochrane Handbook for Systematic Reviews of Interventions which contains random sequence generation, allocation concealment, blindness, incomplete outcome data, selective outcome reporting, and other biases. Additionally, each of the aspects was ranked low risk of bias, high risk of bias, and unclear risk of bias. The evidence grade was assessed using the guidelines of the GRADE (Grading of Recommendations, Assessment, Development, and Evaluation) working group including the following items: risk of bias, inconsistency, indirectness, imprecision and publication bias. The recommendation level of evidence was classified into the following categories:

(1) high, which means that further research is unlikely to change confidence in the effect estimate;
(2) moderate, which means that further research is likely to significantly change confidence in the effect estimate but may change the estimate;
(3) low, which means that further research is likely to significantly change confidence in the effect estimate and to change the estimate; and
(4) very low, which means that any effect estimate is uncertain. GRADE pro Version 3.6 software is used for the evidence synthesis.

### 2.6 Statistical analysis

The risk differences with 95% confidence intervals (CI) were calculated for dichotomous data, and the weighted mean difference (WMD) with 95% CI was calculated for the continuous data. Heterogeneity between the studies was assessed by the *χ*^2^ test (significant level of *P* < .10) and the I^2^ statistic (*I*^2^ > 50% indicating significant heterogeneity). The results were pooled using the fixed-effect model for *P* > .10 and *I*^2^ < 50% or the random-effect model for *P* < .10 and *I*^2^ > 50%. If significant heterogeneity is found, we will try to explore the source of heterogeneity by subgroup analysis. Publication bias was assessed by drawing contour-enhanced funnel plots. When these plots were not obviously asymmetric, we considered that publication bias was absent. All calculations were carried out with Stata 11.0 (The Cochrane Collaboration, Oxford, United Kingdom).

## 3 Discussion

Cognitive dysfunction is a major consequence of renal dysfunction and has further detrimental effects on quality of life for ERSD patients.^[11]^ Cognitive dysfunction can affect the individual's ability to carry out daily life activities, make decisions, keep appointments, manage belongings, prepare meals, adhere to medications, chose between treatment modalities, and recall recent events.^[12,13]^ There is sufficient evidence linking cognitive dysfunction to renal dysfunction and many nephrologists worldwide have incorporated cognitive dysfunction evaluation as part of the routine evaluation of dialysis subjects.^[14]^ However, the differential impact of dialysis modalities on the course of cognitive dysfunction remains insufficiently explored. Some studies have reported better cognitive dysfunction in peritoneal dialysis compared with hemodialysis patients, indicating that peritoneal dialysis is more beneficial in the management of cognitive impairment, more adequate in reversing uremic encephalopathy, and superior in restoring cognitive capacity.^[15,16]^ However, the evidence is limited by poor study design and small sample size. Our review process will be very rigorous and this article is a protocol of the systematic review and meta-analysis, which presents the detailed description of review implement. The results of our review will be reported strictly following the PRISMA criteria and the review will add to the existing literature by showing compelling evidence and improved guidance in clinic settings.

## Author contributions

**Conceptualization:** Yongjian Ji.

**Data curation:** Yongjian Ji.

**Funding acquisition:** Yinghui Wang.

**Writing – original draft:** Tongbin Gao.
